# Isolation and Characterisation of a Human-Like Antibody Fragment (scFv) That Inactivates VEEV *In Vitro* and *In Vivo*

**DOI:** 10.1371/journal.pone.0037242

**Published:** 2012-05-30

**Authors:** Torsten Rülker, Luzie Voß, Philippe Thullier, Lyn M. O' Brien, Thibaut Pelat, Stuart D. Perkins, Claudia Langermann, Thomas Schirrmann, Stefan Dübel, Hans-Jürgen Marschall, Michael Hust, Birgit Hülseweh

**Affiliations:** 1 Wehrwissenschaftliches Institut für Schutztechnologien (WIS) – ABC-Schutz, Munster, Germany; 2 Technische Universität Braunschweig, Institut für Biochemie und Biotechnologie, Braunschweig, Germany; 3 Centre de Recherche du Service de Santé des Armées (CRSSA-IRBA), La Tronche, France; 4 Defence Science and Technology Laboratory, Biomedical Sciences Department, Porton Down, Salisbury, Wiltshire, United Kingdom; Blood Systems Research Institute, United States of America

## Abstract

Venezuelan equine encephalitis virus (VEEV) belongs to the Alphavirus genus and several species of this family are pathogenic to humans. The viruses are classified as potential agents of biological warfare and terrorism and sensitive detection as well as effective prophylaxis and antiviral therapies are required.

In this work, we describe the isolation of the anti-VEEV single chain Fragment variable (scFv), ToR67-3B4, from a non-human primate (NHP) antibody gene library. We report its recloning into the bivalent scFv-Fc format and further immunological and biochemical characterisation.

The scFv-Fc ToR67-3B4 recognised viable as well as formalin and ß-propionolactone (ß-Pl) inactivated virus particles and could be applied for immunoblot analysis of VEEV proteins and immuno-histochemistry of VEEV infected cells. It detected specifically the viral E1 envelope protein of VEEV but did not react with reduced viral glycoprotein preparations suggesting that recognition depends upon conformational epitopes. The recombinant antibody was able to detect multiple VEEV subtypes and displayed only marginal cross-reactivity to other Alphavirus species except for EEEV. In addition, the scFv-Fc fusion described here might be of therapeutic use since it successfully inactivated VEEV in a murine disease model. When the recombinant antibody was administered 6 hours post challenge, 80% to 100% of mice survived lethal VEEV IA/B or IE infection. Forty to sixty percent of mice survived when scFv-Fc ToR67-3B4 was applied 6 hours post challenge with VEEV subtypes II and former IIIA. In combination with E2-neutralising antibodies the NHP antibody isolated here could significantly improve passive protection as well as generic therapy of VEE.

## Introduction

Venezuelan equine encephalitis virus (VEEV) belongs to the *Alphavirus* genus within the Togaviridae family and was first isolated from horses in the 1930s [1], [2]. Besides equids, several species of this virus family are also pathogenic to man and are recognized as potential agent of biological warfare and biological terrorism. VEEV is listed as a “Dirty Dozen agent” and is classified as Category B agent by the Centers for Disease Control and Prevention, Atlanta (http://emergency.cdc.gov/agent/agentlist-category.asp). The virus is highly infectious by the aerosol route [3] and an intentional release as a small-particle aerosol may be expected to infect a high percentage of individuals within an area of a least 10,000 km^2^ [4]. Moreover, VEEV is responsible for VEE epidemics that occur in South and Central America [5]–[7]. It is a single stranded positive-sense RNA virus and is maintained in a cycle between rodents and mosquitoes in nature. VEEV represents a complex of viruses previously classified as subtypes I to VI. However, recent taxonomic changes have classified only the subtype I viruses as VEEV and differentiate five distinct variants (IA/B, IC, ID, IE, IF; http://ictvonline.org). Mainly the subtypes IA/B, IC and ID have been proven to be pathogenic for man. The disease they cause, ranges from mild febrile reactions to fatal encephalitic zoonoses and outcomes are significantly worse especially for young and elderly patients. Subtypes II–VI are now classified as distinct species (http://ictvonline.org) and especially Everglades and Mucambo virus (formerly subtypes II and IIIA) share a high level of genetic homology to VEEV and cause a similar human disease that may lead to encephalitis and death in a small proportion of cases [8].

Continued effort has been made to develop highly-sensitive monoclonal antibodies as well as recombinant antibodies for the immunological detection and diagnosis of VEEV [9]–[15]. Moreover, different well established identification principles like for example colorimetry, electrochemoluminescence and fluorescence immunoassays have been evaluated for the detection of VEE viruses [9]–[11], [16]–[20].

Two live, attenuated vaccines, TC-83 [21] and V3526 [22] were developed to prevent disease caused by VEEV, Everglades virus and Mucambo virus [23]–[27] but both formulations caused unacceptable levels of reactogenicity to allow for general licensure [23], [28], [29]–[32].

A rather uncertain alternative to live attenuated vaccines are formalin inactivated vaccines against viral equine encephalitis. These vaccines do not produce any adverse side effects but have the disadvantage that they are of limited potency and available for humans at high risk only. The formalin inactivated VEEV vaccine, C84, for example, provides only a limited protection from aerosol challenge. It induces a limited neutralisation antibody response and requires regularly and periodic boosters [26]. Therefore, antiviral therapies effective in prophylaxis and treatment of VEEV infection are required and the administration of virus neutralising or otherwise inactivating antibodies could serve as a reasonable alternative to vaccination. In addition, the application of murine antibodies to humans is often critical and limited due to their high immunogenicity, risk of serum sickness and anaphylactic shock. Therefore, human or humanised antibodies as well as antibodies from non-human primates like macaque could offer an alternative for passive protection or therapeutic treatment of VEE.

In this work, we describe the isolation of the anti-VEEV single scFv ToR67-3B4 using antibody phage display from a non-human primate (NHP) antibody gene library. We describe the immunological and biochemical characterisation of its cognate Fc-fusion and investigate its *in vivo* stability as well as its *in vivo* and *in vitro* inactivation potential in BALB/c mice.

## Materials and Methods

### Ethical Statement

#### Macaque care

The animal experiment was approved by the ethics committee for animal experimentation of IRBA-CRSSA (Institut de Recherche Biomédicale des Armées - Centre de Recherche du Service de Santé des Armées). The ethics committee number for the VEE macaque immunisation was 2008/03.1. The immunisation with inactivated virus particles was performed according to the governmental french ethical guidelines: “Partie reglementaire du livre II du code rural (Titre I, chapitre IV, section 5, sous section 3: expérimentation sur l'animal)”, “Décret 87–848 du 19-10/1987 relatif aux expériences pratiquées sur les animaux vertébrés, modifié par le décret 2001/464 du 29/05/2001”, “Arrêté du 29 octobre 1990 relatif aux conditions de l'expérimentation animale pour le ministère de la défense” and “Instruction 844/DEF/DCSSA/AST/VET du 9 avril 1991 relative aux conditions de réalisation de l'expérimentation animale”. Animal care procedures were in compliance with the regulations detailed under the Animal Welfare Act [33] and in the Guide for the Care and use of Laboratory Animals [34]. Animals were kept under stable temperature (22±2°C) and relative humidity (50%) conditions, with 12 hours of artificial lighting per day. Their cages were individual (6 per room) and each had a perch. Animals were fed twice a day, once with dried food, once with fresh fruits and vegetables, and water was provided concurrently. The food intake and general behaviour were observed during feeding by animal technicians, who were able to access a veterinarian if needed. Systematic veterinary visits to each NHP-room were conducted twice a week. General anesthesia was administered prior to any blood or bone marrow collection. Additionally, subsequent analgesics were administered in the following days by animal technicians if pain was suspected, according to the observations of animal behaviour.

#### Mice infection

The use of mice for passive protection studies for research purposes complied with UK guidelines. Animal protocols adhered to the Animals (Scientific Procedures) Act 1986, and protocols were approved by the UK Home Office. The study was performed under project licence 30/2425 and was approved internally by a Defence Science and Technology Laboratory (Dstl) ethical review process.

#### Cell culture and virus production

All viruses used in this study represent models for biowarfare agent relevant Alphavirus species and are either part of the strain collection of the Armed Forces Scientific Institute for Protection Technologies – NBC Protection (WIS) or are part of the strain collection of the Defence Science and Technology Laboratory, Porton Down, UK. Strains of VEEV from subtypes IA/B (Trinidad donkey; TrD), IC (P676), ID (3880), IE (Mena II), IF (78V), Everglades virus (formerly subtype II, strain Fe37c), Mucambo virus (formerly subtype IIIA, strain BeAn8), Pixuna virus (formerly subtype IV), Cabassou virus (formerly subtype V, strain CaAr508) and Rio Negro virus (formerly subtype VI, strain AG80) were kindly supplied by Dr. R.E. Shope (University of Texas Medical Branch, USA). Eastern equine encephalitis virus (EEEV) strain H178/99 and Western equine encephalitis virus (WEEV) strain H160/99 used in this study were received from the National Collection of Pathogenic Viruses (NCPV), UK.

Alphaviruses were either propagated in Baby Hamster Kidney (BHK), Vero-B4 (African green monkey kidney) or L929 (murine fibroblast) cells at 37°C and 5% CO_2_ in a biosafety 3 facility according to standard procedures as described [18], [35]–[37]. The cell lines were obtained from the DSMZ-ACC 33 (Deutsche Sammlung von Mikroorganismen und Zellkulturen GmbH), Braunschweig, Germany or the European Collection of Animal Cell Cultures, Porton Down, Salisbury, UK.

All viruses were harvested from infected cells when 50–75% of the cell monolayer showed evidence of viral cytopathic effect (CPE). Virus titers were either determined by the 50% tissue culture infective dose (TCID_50_/mL) method according to Spearman and Kaerber or plaque assay [38]–[40]. Titers typically ranged from 1.0×10^7^ to 1.2×10^10^ TCID_50_/ml.

#### Purification of Alphaviruses

Virus containing supernatants from infected Vero cells were either purified by affinity chromatography on Matrex Cellufine Sulfate Medium™ (Virus Recovery System, VRS, Chisso America Inc., NY, USA) or by isopycnic density gradient centrifugation as described below. Matrex Cellufine Sulfate Medium™ (VRS) is a cellulose bead medium functionalised with a low concentration of sulfate esters that operates similar to a cation-exchange resin and has a high affinity for enveloped viruses. It selectively adsorbs complete virus particles as well as viral coats according to their charge. Briefly, 50 mL resin was equilibrated with adsorption buffer (0.01 M phosphate buffer, pH 7.5). Up to 200 mL of virus containing prefiltered cell culture supernatant was loaded onto the column which then was washed twice with 0.01 M phosphate buffer, pH 7.5. Elution of virus particles was performed with 1 M NaCl.

Purification of virus particles was carried out in two steps. Prepurification was performed by the sucrose cushion ultracentrifugation method (20% sucrose cushion), which causes low mechanical stress and allows the concentration and collection of morphologically intact particles after centrifugation at 112,000× g for 2 to 3 hours. The pellet was resuspended in 0.5 to 1 mL phosphate buffered saline (PBS) and further purified by isopycnic density gradient centrifugation (20 to 60% sucrose) for 18 hours at 217,500× g. The virus containing fraction was removed and stored at −80°C until subjected to further analysis.

#### Inactivation of Alphaviruses

Inactivation of all viral antigens was performed with a final concentration of 0.1% ß-propionolactone (ß-PL, Sigma-Aldrich, Taufkirchen, Germany). Immediately before use a 10% ß-PL solution was prepared and 0.1 ml of this dilution was incubated with 5 ml of virus containing cell culture supernatant (pH 8 to 8.5) for 1 h at 4°C and 4 hours at 37°C with constant stirring. After 2 and 4 hours the pH of the cell culture supernatant was controlled and adjusted if necessary. In order to achieve a complete hydrolysis of remaining ß-PL after the inactivation the supernatant was kept at 4°C for another 12 to 18 hours. After low speed centrifugation of the sample for 30 min at 1000× g the viral inactivation was verified by inoculation of Vero cells and monitoring for cytopathic effects (CPE) for 3 to 5 days.

#### Animal immunisation

A male cynomolgus macaque (Macaca fascicularis) was immunised by intramuscular injection with 100 µl of the commercially available veterinary vaccine Fluvac Innovator Triple EFT plus EHV (Fort Dodge Animal Health, USA). This all-around vaccine is normally applied to horses in order to protect them from equine encephalitis caused by EEE, WEE and VEE viruses. In addition, the vaccine contains inactivated rhino- and influenza viruses as well as tetanus toxoid to protect horses from equine rhinopneumonitis, influenza and tetanus.

days after this first immunisation the macaque was immunised with 100 µl of VRS purified ß-PL inactivated VEEV TC83 (TCID_50_/ml of 2×10^10^) in combination with complete Freund's adjuvant. Subsequently, 5 additional antigen injections of 100 µl were performed after 65, 90,150, 210 and 350 days with incomplete Freund's adjuvant. Ten days after the final antigen injection the immune library was generated.

After each boost immune sera were gathered, inactivated for 20 min at 56°C and tested for VEEV specific antibody titer by ELISA. For this purpose either VRS-purified culture supernatants from VEEV TC83 infected Vero cells or uninfected cells were immobilised on 96 well-microtiter plates (Maxisorp™, Nunc, Wiesbaden, Germany). For the determination of VEEV specific antibody titers serial dilutions of pre-immune-serum (PI 543) and immune sera (S543) were applied. Specifically bound antibodies were detected by incubation with rabbit anti-monkey IgG conjugated to horseradish peroxidase (1∶10.000, Sigma-Aldrich, Taufkirchen, Germany) for 30 minutes at room temperature. Staining was performed with 3-3′, 5-5′ -tetramethylbenzidine (TMB, Serva, Heidelberg, Germany) and stopped with 2 M sulphuric acid after 10 minutes. Absorbance was measured at 450 nm.

#### Library construction and screening of recombinant antibodies

Lymphocytes of the macaque bone marrow were sampled after 6 boosts and subjected to RNA preparation by using Tri Reagent (Molecular Research Center Inc, Cincinnati, USA). The isolated RNA was reverse transcribed to cDNA and diverse combinations of forward and reverse primers were used to amplify DNA, coding for the variable regions of antibodies, VLκ and VH [41]. In order to obtain the cognate sublibraries, the PCR fragments were subcloned into the pGemT vector (Promega, Madison, Wisconsin) as described [41]–[45]. For the phage-displayed library the precloned VH and VL repertoire was reamplified and specific restriction sites were inserted by PCR [42]. For the construction of the library the reamplified VH and VL genes were subcloned into the phagemid pHAL14 in two successive ligation steps. The VH PCR products were subcloned into pHAL14 as *Hind*III-*Nco*I fragment, the VL PCR products were inserted as *Not*I-*Mlu*I fragment.

The phagemid pHAL14 was chosen as library vector because it allows phage display and the production of soluble antibody fragments without the necessity of recloning. pHAL14 is a high-copy plasmid and contains the scFv under the control of the lacI promoter/operator region which is inducible by IPTG. A His- and c-Myc-tag are encoded at the C-terminus in frame with the scFv and allow the straightforward purification of the recombinant antibody [45].

All cloning and ligation procedures necessary for the library construction were performed in *E. coli* XL1-Blue MRF' (Stratagene, Amsterdam, The Netherlands) according to Sambrook et al., 1989 [46]. For DNA plasmid and PCR product purification the commercial Plasmid Mini Kit and QIAquick PCR Purification Kit from QIAGEN, Hilden, Germany, were used.

The library was packaged by using Hyperphage [47], [48]. Therefore, 1 ml of the antibody gene library stock was grown to an OD600 nm of 0.4 to 0.5 and about 1.25×10^10^ *E. coli* XL1-Blue MRF' transformants were infected with 2.5×10^11^ Hyperphage.

*E. coli* cells and Hyperphage were incubated for 30 min at 37°C without shaking, followed by a further incubation for 30 min with shaking at 250 rpm. The infected cells were harvested by centrifugation and transferred to glucose-free TY-medium containing 100 µg/ml ampicillin and 50 µg/ml kanamycin in order to repress the lac promoter of pHAL14 and allow the expression of the scFv::pIII fusions. After incubation overnight at 250 rpm and 30°C bacteria were removed by centrifugation and phage particles in the culture supernatant were precipitated as previously described [49]. The precipitated and packaged antibody phage library was then resuspended, titered and stored at 4°C until used for the panning and screening [49].

Surface presentation onto phage of the library was examined by SDS-PAGE, Western blotting and anti-pIII immuno-staining as described [50].

The process for the selection of specific scFv from a phage-displayed library is referred to as “panning,”and, in principle, involves the selection of Abs on the basis of their affinity.

The panning of VEEV specific scFv was performed on VRS purified VEEV presented by specifc mAbs coated onto 96 well microtitre plates (Maxisorb, Nunc, Wiesbaden, Germany) with minor modifications according to [12].

The panning and amplification of specifically bound phage was repeated three times before proceeding with postpanning analysis and each panning included a preselection and selection step. In order to remove unspecific binders, the library was preincubated in parallel with non-infected Vero cells and a nonspecific murine IgG. Therefore, 100 µl of the phage library was incubated for 1 h at room temperature (RT) onto a mixture of coated and concentrated VRS supernatant from non-infected Vero cells plus 1% BSA (bovine serum albumin) in PBST (PBS plus 0.01% Tween 20) containing 10 µg/ml of the murine IgG D1-4G2-4-15 [51].Washing and reamplification of specifically bound phage was performed as described [12].

#### Sequencing and sequence analysis

Sequencing was performed by GATC Inc. (Konstanz, Germany) using the oligonucleotide primer MHLacZ-Pro_f (5′ ggctcgtatgttgtgtgg 3′). The antibody gene fragments were analysed by using VBASE2 (http://www.vbase2.org) and Kabat's database of sequences of immunological interest (www.bioinf.org.uk) as previously described [45], [52].

#### Construction of stable eukaryotic tranfectants, expression and production of scFv-Fc ToR67-3B4

VEEV specific scFv gene fragments were subcloned from the immune library vector pHAL14 into the mammalian expression vector pCMV2.5-hIgG1-Fc by using the restriction sites *Nco*I and *Not*I [49]. The mammalian expression vector allows the expression of scFvs in frame with the human IgG1 gene. The fusion is under the control of the CMV promoter. Moreover, the vector contains besides the *bla*-gene a neomycin phosphotransferase (Neo) expression cassette for antibiotic selection with G418.

For the stable production of VEEV specific scFv-Fc fusion proteins, Chinese hamster ovary K1 cells (CHO-K1), from the American Type Culture Collection, (ATCC, Rockwell, MD, No. CCL61) were transfected by using 80 µl Polyfect® (Qiagen GmbH, Hilden, Germany) plus 4 to 5 µg of plasmid DNA. Stable clones were selected for resistance to the aminoglycoside antibiotic Geneticin (G418). For this purpose CHO-K1 cells were cultivated to 60% to 80% confluence overnight in 1000 mm^2^ Petri dishes (Nunc, Vienna, Austria) in non-selective Dulbecco's Modified Eagle's Medium (DMEM/HAM's F-12), a nutrient mixture supplemented with L-glutamine and sodium bicarbonate, containing 10% (v/v) fetal calf serum (FCS) and 0.1% (w/v) penicillin and 0.1% (w/v) streptomycin (PAA, Parsing, Austria) at 37°C in 4% CO_2_ atmosphere. In order to support the DNA-lipid complex formation, plasmid DNA and Polyfect® were preincubated in serum- and antibiotic-free medium for 10 minutes at room temperature prior to transfection. While the lipid-DNA complex formation took place, CHO-K1 cells were washed with PBS. Afterwards, 7 ml of medium with FCS and penicillin/streptomycin was added to the complex. The whole mixture was then immediately added to the washed cells by gently swirling. Cells were incubated for 3 h with the transfection complex. Subsequently the medium was removed and replaced with fresh non-selective DMEM/HAM's F-12 containing 10% (v/v) FCS and 0.1% (w/v) penicillin/streptomycin before the plates were incubated overnight at 37°C in 4% CO_2_. A further medium change was performed one day after the transfection. The transfected cells were trypsinised and re-sown 1∶20 or 1∶50 in new Petri dishes on selective medium, containing additionaly 700 mg/ml G418. Medium change was performed every 3 to 4 days and first stable and G418-resistant clones were visible about 3 weeks after transfection. Four and five weeks after transfection single clones were isolated and cultivated onto 24 well plates (Nunc, Vienna, Austria). Since the productivity of individual clones varies, we tested the supernatant of 48 clones for VEEV-specific scFv-fusion production in ELISAs as described. Finally, three of the best expressing CHO-K1-clones were cultivated as suspension culture in a miniPerm bioreactor (Sarstedt, Nümbrecht, Germany) in DMEM/HAM's F-12 containing 10% (v/v) (FCS), 1% (w/v) penicillin/streptomycin and 700 mg/ml G418 at 37°C in 4% CO_2_. The culture supernatant was harvested twice a week and replaced by fresh medium. Purification of recombinant fusion proteins was done by immunoaffinity chromatography on goat anti-human (GAH) sepharose.

#### Biotinylation of mAbs and scFv-Fc fusions

One to two mg of mAb or scFv-Fc was dissolved in sodium bicarbonate buffer, pH 8.5, and incubated with an aliquot of the biotin N- hydroxysuccinimide ester (long arm, water soluble) from Vector laboratories, CA, USA, equal to 1/10 the weight of the protein to be labeled. The mixture was incubated for 2 hours at room temperature. In order to stop the reaction 10 mg of glycine was added and unreacted biotin was removed by gel filtration with PD-10 desalting columns containing Sephadex G25 (GE Healthcare, USA) according to the suppliers' protocol.

### Enzyme-linked immunoassays (ELISAs) with scFv-Fc fusions

#### Indirect ELISA

Sucrose density gradient-purified antigen from strains TrD, P676, 3880, Mena II, 78 V, Fe37c, BeAn8, Pixuna, CaAr508 and AG80 (subtypes IA/B, IC, ID, IE, IF, formerly subtypes II IIIA, IV, V and VI) were coated on 96-well Maxisorp™ microtiter plates purchased from Nunc, Wiesbaden, Germany. The viral protein concentration was determined by SDS-PAGE and scanning dosimetry, dilutions were performed in 50 mM bicarbonate buffer pH 8.5. After blocking with PBS containing 1% (w/v) skimmed milk powder (Sigma-Aldrich, UK) plus 0.1% (v/v) Tween 20 detection of VEEV strains was performed with different log_10_ dilutions of scFv-Fc ToR67-3B4 for 2 hours at room temperature (RT). The starting concentration was 1 µg/ml. Subsequently the microwell plates were washed with PBS-T and the presence of bound antibody was detected by using peroxidase-conjugated goat-anti-human IgG (1∶4.000, AbD Serotec, UK) as secondary antibody. TMB-substrate (Sigma-Aldrich, UK) was used as chromogenic substrate, absorbance was measured at 450 nm.

#### Direct Sandwich ELISA

All sandwich ELISAs were performed in 96 microwell plates (Maxisorp™, Nunc, Wiesbaden, Germany) that were either coated with 3 µg/ml antibody or scFv-Fc by incubation for 2 h at 37°C or overnight at 4°C. For the detection of VEEV we used either the monoclonal antibody (mAb) VEE-WIS1 or a mixture of mAbs VEE-WIS1, SFV3/4 and SFV 12/2 as capture antibodies [9], [16]–[19]. MAb VEE-WIS1 (E1-specific) regognises specifically VEEV of subtype IA/B, while the antibody mixture binds Old world as well as New world Alphaviruses. In order to capture WEEV specifically mAb SVF 12/2 was applied. The ELISA to detect EEE virus was done on plates coated with a mixture of mAbs SFV 8/6 (E1-specific), SFV3/4 and SFV 12/2 [9], [16]–[19]. Subsequently antibody coated microtiter plates were washed and blocked with 1% FCS in PBS-T during 1 h at room temperature. After further washes, wells were additionally overlayed with liquid plate sealer (Candor Bioscience GmbH, Wangen, Germany), stored at 4°C and used for ELISA studies within three to six weeks. Samples with virus were incubated for 2 h at room temperature on precoated microwell plates and afterwards extensively washed. Bound viruses were detected by using biotinylated scFv-Fcs or mAbs in appropriate dilutions (1∶500, 1∶2000 or 1∶5000) and incubation for 1 h at 37°C. After three further washes the conjugate Streptavidin-horseradish peroxidase (PSA, GE Healthcare, USA) was applied to the wells in a 1∶ 6000 dilution and plates were incubated for 30 min at RT with agitation. Bound biotinylated mAbs and fusions were detected by using TMB staining (commercial solution from Serva, Heidelberg, Germany) that was stopped with 2 M sulphuric acid after 10 minutes. Absorbance was measured at 450 nm.

#### Immunoblot analysis

ScFv-Fc fusion proteins and several monoclonal antibodies as positive controls were used to detect VEEV specific envelope proteins.

Therefore, purified VEEV particles were disrupted either by incubation for 20 minutes at 56°C or 5 minutes at 95°C in Laemmli sample buffer [53] containing either no or 1 mM DTT. The viral proteins were separated by 15% SDS-PAGE and blotted onto a nitrocellulose membrane (Protran, BA85, Sigma-Aldrich, Taufkirchen, Germany). The membrane was blocked with PBS plus 1% Casein, pH7.4 (Thermo Fisher Scientific GmbH, Dreieich, Germany) for 1 h at RT. For the detection of virus-specific proteins, the membrane was incubated with either biotinylated scFv-Fc (Tor67-3B4: 1∶5000) fusions or mAbs (SFV 8/6: E1-specific mAb, 1∶10.000; WIS-VEE1: E1-specific mAb, 1∶1.000; 1A3B7: E2-specific mAb, 1∶1.000) for 1.5 h at RT in the dark. After 3 washes with PBS-T the membrane was incubated with the conjugate streptavidin-alkaline phosphatase (1∶1000, GE Healthcare, Munic, Germany) for 30 min. Staining and visualisation of specific proteins was performed with 5-Brom-4-Chlor-3-Indolyl-Phosphat/Nitro Tetrazolium Blue Chloride (NBT-BCIP, Thermo Fisher Scientific GmbH, Dreieich, Germany) according to standard procedures.

#### Immunohistochemistry

Vero cells (100 µl of 2×10^5^ cells/ml) were grown in 96-well microtiter plates for 24 h and subsequently infected with serial log_10_ dilutions of either VEEV TC83 or TrD. Non-infected Vero cells served as negative control. Specific staining of VEEV infected cells was performed with biotinylated scFv-Fc ToR67-3B4 and mAb SFV 8/6 as positive control. In detail, one day post infection the infected cells were fixed with 3% formalin in PBS for 3 h at 4°C. Afterwards the fixed samples were incubated with their cognate antibodies in either a 1∶5000 or 1∶10.000 dilution in PBSF-T (PBST plus 1% FCS) for 1 h in a humid chamber at 37°C. After 3 consecutive washes in PBS-T the fixed cells were incubated for 30 min with the conjugate streptavidin - horseradish peroxidase (PSA, GE Healthcare, USA) diluted 1∶6000 in PBS-FT. After further extensive washing and in order to visualise infected cells microscopically, the samples were incubated with the precipitating colorimetric peroxidase substrate TMB (KPL, Germany) for 10 minutes. Staining was stopped by rinsing the plates with Millipore-purified water.

### Neutralisation tests

#### Neutralising peroxidase-linked antibody (NPLA) assay

The NPLA assay for scFv-Fc fusions and complete IgG antibodies was done according to the technique of Jensen [54] with several modifications. All assays were performed in 96 well microwell plates and Vero cells were used as host for infection. Fifty µl of two-fold dilutions of scFv-Fc fusions or mAbs (starting concentration between 0.5 to 1.0 mg/ml) were incubated with an equal volume of VEE virus strains TC-83, TrD or Fe3-7c with a TCID_50_/ml of 5×10^4^ for 2 hours at 37°C in 96-well plates. Following this incubation, 100 µl of freshly trypsinised Vero cells at a concentration of 4×10^5^ cells/ml were transferred to the antibody virus mixture.

As positive control for infection, virus samples not preincubated with any antibody as well as virus samples preincubated with VEEV specific antibodies that do not display any neutralising activity like mAb WIS VEE1 were used. Moreover, virus samples preincubated with mAb SFV 8/6 and 1A3B-7, both antibodies with known neutralizing capacity ([55] and Hülseweh, personal communication) were applied as a positive control and non-infected Vero cells served as negative control. Twenty to twenty-four hours post infection all cell monolayers were fixed with 3% formalin in PBS for 3 h at 4°C and infection of cells was demonstrated by specific immunostaining of viral antigens. After washing with PBS-T, the monolayers were overlaid with 100 µl of a 1∶5000 dilution of the VEEV-specific biotinylated mAb SFV 8/6 and incubated for 2 h at RT. Bound biotinylated mAbs were detected by using the streptavidin HRP conjugate (GE Healthcare, USA, 1∶6.000) and visualisation was performed with precipitating TMB as substrate. The staining reaction was stopped by adding 100 µl of 1 M sulphuric acid and absorbance was measured at 450 nm in a microwell plate reader. The absorbance values were correlated to relative infectious activity. Thereby absorption at 450 nm (OD450 nm) values of the positive control, namely Vero B4 cells infected with VEEV, were set as 100% infectivity. A reduction equal or greater than 50% in A450_nm_ in the wells was considered as indicative for neutralisation.

#### Plaque reduction Assay

The ability of scFv-Fc ToR67-3B4 (25 µg) to neutralise virus infectivity was determined by mixing the construct with VEEV strains TrD, Fe37c or BeAn8 (approximately 100 pfu) and incubating at 4°C overnight. Residual infectious virus was estimated by a standard plaque assay in L929 cells as described [37].

#### Determination of the in vivo half-life of scFv-Fc ToR67-3B4

Sera were harvested from the marginal tail veins of three BALB/c mice (6–8 weeks old; Charles River Laboratories, UK). Five days later, 100 µg scFv-Fc ToR67-3B4 were injected by the intraperitoneal route. Serum samples were collected 8 h, 1 d, 3 d, 5 d, 10 d, 15 d and 25 d post-administration. The concentration of scFv-Fc ToR67-3B4 in sera was determined by ELISA using sucrose density gradient-purified β-propiolactone inactivated antigen of VEEV strain TC-83. Antibody concentrations were estimated by comparison of the absorbance values generated by diluted serum samples with a standard curve prepared with scFv-Fc ToR67-3B4.

#### Statistical methods

Statistical analysis was performed using GraphPad Prism software (http://www.graphpad.com). To determine the half-life of scFv-Fc ToR-3B4 *in vivo*, a linear regression model was fitted to the log transformed data to estimate the rate of change over time. A change of 0.30 units on the log scale equates to a 50% reduction of the data on the original scale. This value is divided by the estimated model slope to obtain the half-life.

#### Passive protection studies

BALB/c mice, 6–8 weeks old, were used for all passive antibody protection studies. Mice were challenged by the airborne route with 100LD50 strain TrD [56], [57]. The LD50s for strains Mena II, Fe37c and Mucambo virus have not been determined. However, titres were used in the collision nebuliser that have reliably achieved 100% mortality in previous experiments [36], [37]. Afterwards mice remained either untreated or were injected intraperitoneally with 100 µg scFv-Fc ToR67-3B4, human IgG (Sigma-Aldrich, Taufkirchen, Germany) or control construct (anti-WEEV). Mice received just one injection at a single time-point (6, 24, 48 or 72 h) post-challenge and were monitored for clinical signs of infection (piloerection, hunching, inactivity, excitability and paralysis) twice daily by an observer who was unaware of the treatment allocations. Animals were scored according to the severity of the clinical signs and were humanely culled as appropriate [58]. These experiments therefore record the occurrence of severe disease rather than mortality. Even though it is rare for animals infected with virulent VEEV and showing signs of severe illness to survive, our use of humane endpoints should be considered when interpreting any virus dose expressed here as 50% lethal doses (LD_50_). In one experiment, sera were harvested by cardiac puncture from mice that were alive fourteen days post-challenge. Samples were assayed for VEEV-specific antibodies using sucrose density gradient-purified β-propiolactone-inactivated antigen from strain TC-83.

## Results

### Construction of the macaque immune library and screening of VEEV specific recombinant antibodies

**Figure 1:**
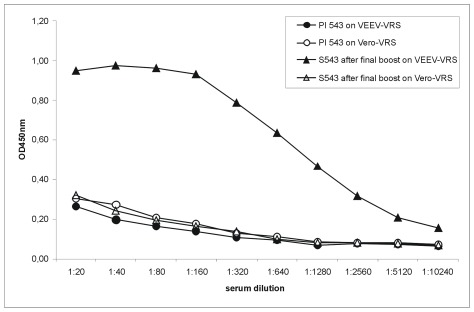
Titration of monkey sera after the final boost. Either VRS-purified culture supernatants from VEEV TC83 infected Vero cell or uninfected cells (negative control) were immobilised on 96well-microtiter plates. For the detection of a VEEV specific antibody titer, serial dilutions of pre-immune serum (PI 543) and immune serum (S543) after the sixth boost were applied. Bound antibodies were detected by rabbit anti-monkey IgG conjugated to horseradish peroxidase and staining with TMB.

In order to generate antibody fragments reactive to members of the VEE virus sero-complex we constructed a VEEV specific scFv antibody gene library 360 days post the sixth immunisation of a non-human primate (Macaca fascicularis) with ß-PL inactivated VEEV TC83. Donor sera ELISA titers to VEEV TC83 rose continuously in the course of immunisation and showed a clear VEEV-specific binding in comparison to the pre-immune serum. After six VEEV TC83 injections, the specific serum antibody titer was equal to 1∶10240 (Figure 1). Moreover, this final serum was tested positive for neutralisation of VEEV TC83 (data not shown).

For the macaque immune library, total RNA was isolated from lymphocytes of the bone marrow and reverse transcribed to cDNA. After PCR amplification and precloning of the antibody fragments for VH and VLκ the final phage displayed library was established in the phagemid pHAL14, a vector that allows phage display and the production of soluble antibody fragments without the necessity of recloning. [49]. The library quality was analysed by colony PCR. About 90% of the 1.6×10^7^ independent clones contained a full size scFv insert. In order to improve the antibody display efficiency the library was packaged by Hyperphage, a substitute for M13KO7 helper phage. Correct surface presentation onto phage was examined by SDS-PAGE, Western blotting and anti-pIII immuno-staining as described [50].

All pannings were performed in a biosafety level 3 laboratory by using the VEEV vaccine strain, TC83, as a medically important and epizootic antigen.

The phage display antibody gene library was subjected to 3 rounds of panning and representative phage clones were assessed for their ability to bind to VEEV TC83 immobilized onto 96 microwell plates. The enrichment of false-positive antibody phage particles was excluded by preincubation of the library with the supernatant of non-infected Vero cells. Non-specific binding of scFv phage to the VEEV-specific capture antibodies mAb 8747 and mAb VEE-WIS1 was reduced by preabsorption of the library. Moreover, these two antibodies were added for competitive binding during the panning procedure.

In total, 322 monoclonal soluble scFvs produced from *E. coli* clones were analysed by ELISA on immobilized inactivated VEEV particles. Twenty-four monoclonal ELISA positive binders were identified. Their specific antigen binding was confirmed on infectious as well as on ß-PL inactivated VEEV of subtype IA/B.

### Sequencing and nucleic acid analysis of scFv clones

The DNA fragments of 24 positive monoclonal binders were sequenced in order to identify unique binders and to define the germline gene family and the amino-acid subgroups of the heavy (V_H_) and light (V_L_) chain variable regions. However, three rounds of panning yielded only one unique VEEV-specific clone that predominated the resulting antibody panel. All binders were identical in their V_H_ and V_L_ sequences. Thereafter, further analysis was exclusively performed with the binder ToR67-3B4. The alignment of the amino acid sequence of ToR67-3B4 to VBASE2, the integrative database of germ-line variable genes from IgG loci of human and mouse, showed the highest similarity to the human IGHV4-59*01 and IGKV2-30*01. Furthermore, we calculated the G-score of the antibody's variable (V) regions since macaque antibodies have been suggested to have extremely human-like character [59], a property that could be especially beneficial for a therapeutical antibody. The V_H_ region of ToR67-3B4 had a G-score of 0.707 thus meaning that it has the same level of similarity as 24% V_H_ sequences present in Kabat's database. If the human V_H_ sequences present in Kabat's database are regarded as representative of human V_H_ sequences, this result may be summarized as “the V_H_ region of ToR67-3B4 is as human as 24% of human V_H_ regions”. The V_L_ chain displayed a G-score of −1.505, indicating that it is “as human as” 7% of human sequences present in the Kabat database.

### Enhancing ToR67-3B4 binding properties by subcloning the scFv in an eucaryotic expression vector

By one step subcloning, the scFv ToR67-3B4 was inserted as a *Nco*I-*Not*I fragment into the mammalian expression vector pCMX2.5-hIgG1-Fc-XP, that allowed the fusion of scFv antibody coding regions to the gene fragment encoding the human IgG1 Fc part (hinge-CH2-CH3) and enabled the stable expression of homodimeric and bivalent scFv-Fc fusion proteins in CHO-K1 cells. Moreover, the IgG1 Fc part allowed routinely high affinity purification with goat anti-human antibodies coupled to sepharose. The fusion of a scFv to a Fc part mediates enhanced *in vitro* protein stability, could prolong serum half-life and mediates IgG effector functions if necessary [60].

### Reactivity of scFv-Fc fusion ToR-3B4 to different VEEV strains and other Alphaviruses

**Figure 2:**
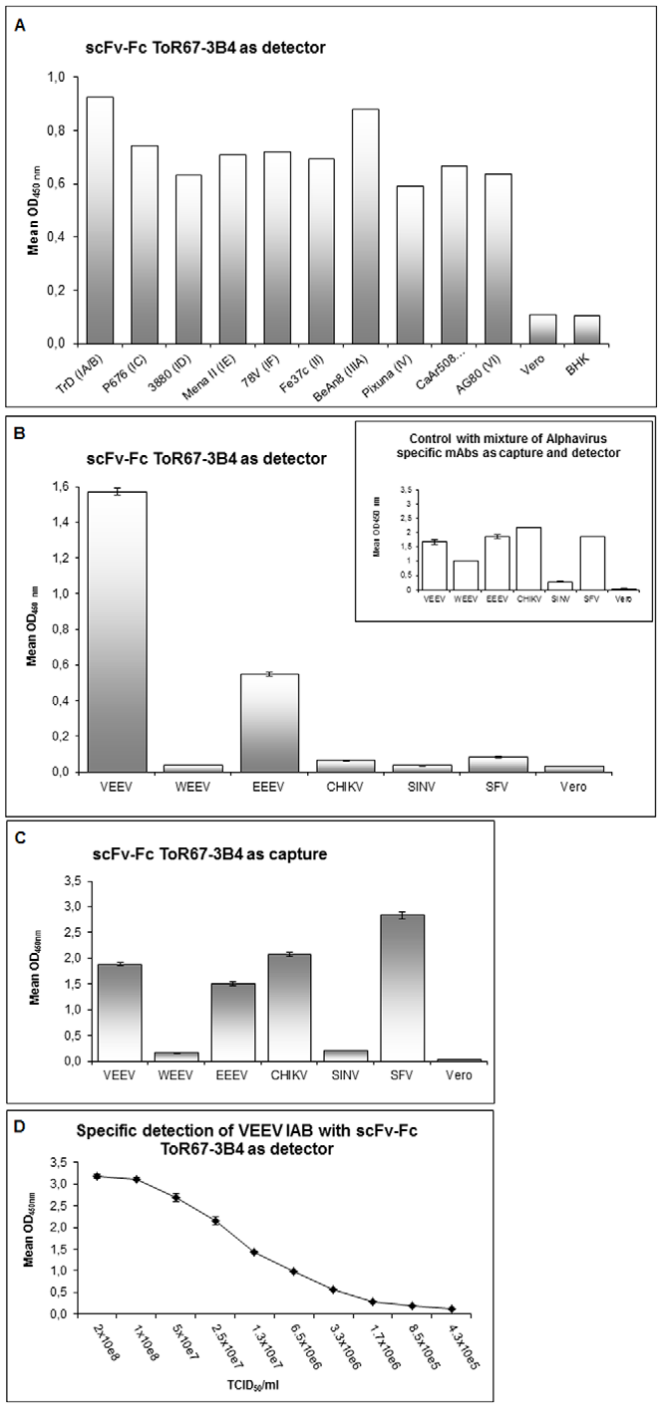
Reactivity of scFv-Fc ToR 67- 3B4 to Alphavirus subspecies and species. Panel **2A** shows the antigen binding efficiency of ToR67-3B4 on different immobilised VEEV subtypes at a concentration of 1 µg/ml, n = 1 for all data points. Panel **2B** illustrates the cross-reactivity of scFv-Fc ToR67-3B4 to a range of Alphavirus species analysed by a sandwich ELISA. Antigens were captured by an anti-Alphavirus mAb mix, specifically bound virus was detected by biotinylated scFv-Fc ToR67-3B4 (1∶5000). The insert in the upper right corner of the bar chart shows the positive control and demonstrates that all Alphaviruses except SINV are specifically captured by the antibody mixture. Detection of virus was performed with the same biotinylated anti- Alphavirus mAb mix. All viral antigens were applied with a TCID_50_/ml of 5×10^7^ to 10^8^. Culture supernatant of non-infected Vero cells was used as negative control. The mean values of two ELISAs from two independent experiments are shown. In panel **2C** scFv-Fc ToR67-3B4 was applied as capture antibody in combination with the cognate Alphavirus-specific antibody mixture for group-specific detection. Viral antigens were applied with a TCID_50_/ml of 5×10^7^ to 10^8^. Culture supernatant of non-infected Vero was used as negative control. The mean values of two ELISAs from three independent experiments are shown. Panel **2D** shows the detection limit for vaccine strain TC83 (subtype IA/B) using capture antibody mAb VEE WIS1 paired with biotinylated scFv-Fc ToR67-3B4 detector antibody (1∶5000). Virus was titrated in 2-fold dilutions. The data represents 2 separate experiments with 3 replicates of each concentration. Abbreviations used in this legend are BHK: Baby hamster kidney cells, VEEV: Venezuelean equine encephalitis virus, WEEV: Western equine encephalitis virus, EEEV: Eastern equine encephalitis virus, CHIKV: Chikungunya virus, SINV: Sindbis virus and SFV: Semliki Forest virus.

The ability of scFv-Fc ToR67-3B4 to recognise different subtypes of VEEV strains was evaluated by an indirect ELISA. For this purpose, sucrose density gradient-purified antigens from strains TrD, P676, 3880, Mena II, 78 V, Fe37c, BeAn8, Pixuna, CaAr508 and AG80 (subtypes IA/B, IC, ID, IE, IF, formerly subtypes II, IIIA, IV, V and VI respectively) were prepared and coated in comparable amounts onto 96-well microtiter plates. At a concentration of 1 µg/ml, scFv-Fc ToR67-3B4 reacted with multiple VEEV subspecies in ELISA with a strong affinity to all subtype 1 varieties (IA/B, IC, ID, IE, IF) but also recognized Everglades, Mucambo, Pixuna, Cabassou and Rio Negro virus (formerly subtypes II, IIIA, IV, V and VI, see Figure 2A). Diluting scFv-Fc TOR-3B4 proved that the recombinant antibody reacted equally well with all the VEEV strains to which it was able to bind strongly.

Moreover, the ability of scFv-Fc ToR67-3B4 to detect different Alphavirus species was tested by a direct sandwich ELISA, using scFv-Fc ToR67-3B4 either as detection (Figure 2B) or capture antibody (Figure 2C). As illustrated in figure 2B scFv-Fc ToR67-3B4 displayed only marginal cross-reactivity to other Alphavirus species except for EEEV if applied for detection. The recombinant antibody can be combined with the capture antibody mAb VEE WIS1 for the highly specific detection of VEEV. At least a TCID_50_/ml of 10^6^ was manifested as detection limit in a sandwich ELISA for strain TC83 (IA/B).

Despite this, if scFv-Fc ToR67-3B4 was applied as capture antibody in combination with an Alphavirus-specific detection mix the recombinant antibody displayed a considerably broader cross-reactivity. As illustrated in figure 2C, scFv-Fc ToR67-3B4 bound additionally to VEEV, EEEV, CHIKV and SFV. It also showed a low relative binding efficiency to SINV and WEEV.

### Immunohistochemistry and Immunoblot analysis

**Figure 3:**
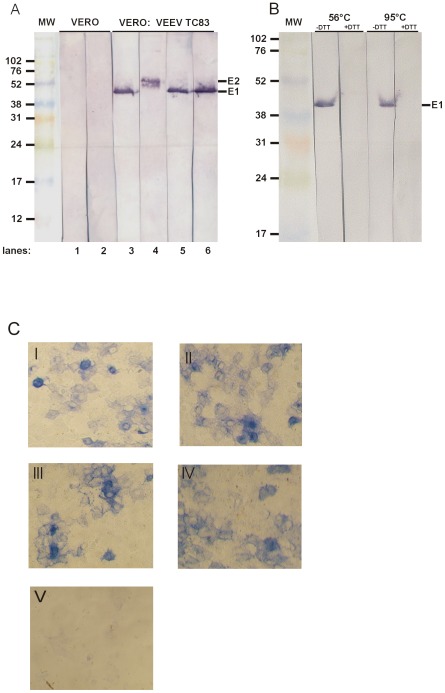
Immunological analysis of VEEV glycoproteins. Panel A and B show the immunoblot analysis of VEEV glycoproteins with scFv-Fc ToR67-3B4 and monoclonal antibodies. Proteins from VEEV strain TC-83 were either resolved on an 8–15% gradient (panel A) or a linear 12% polyacrylamide gel (panel B) under denaturing and non-reducing conditions at 56°C (panel A) or at 56°C and 95°C (panel B) under denaturing conditions with or without 100 mM DTT. After Western blotting the membrane was cut in stripes and was probed with either scFv-Fc ToR67-3B4 (Panel A lane 2 and 6; panel B all lanes) or E1- and E2-specific mAbs (Panel A lane 1 and 5: SFV 8/6 (E1-specific); lane 3: WIS-VEE1 (E1-specific); lane 4: 1A3B7 (E2-specific)). Scale indicates protein size in kDa. Panel C depicts the immunohistochemistry of Vero cells infected with VEEV TrD, 1 day post infection. Photomicrographs were obtained from infected (I–IV) and non–infected (V) cells with a 10fold magnification lens. Cells in panel I and II were stained for VEEV antigen with biotinylated scFv-Fc ToR67-3B4, infected cells in panel III and IV were detected with biotinylated mAb SFV 8/6 as positive control. Non-infected Vero cells in panel V were stained with scFv-Fc ToR67-3B4 as negative control.

ScFv-Fc ToR67-3B4 can be used for immunohistochemistry of VEEV infected cells and Western blot analysis of VEEV proteins (Figure 3). As expected, Vero cells infected with VEEV TrD displayed a strong perinuclear signal 1 day post infection when stained with either scFv-Fc ToR67-3B4 or the well established mAb SFV 8/6 [11], [16], [17]. Similar results were obtained for Vero cells infected with the vaccine derivative TC83 (data not shown) while non-infected cells showed no specific binding.

The glycoprotein envelope of Alphaviruses is made up of two proteins, E1 and E2. While E1 is responsible for the fusion process with the host cell membrane, E2 is responsible for cell receptor binding. In order to examine which VEEV specific structural glycoprotein is recognised by scFv-Fc ToR67-3B4 we electrophoretically separated purified virus samples after disintegration in a 15% PAA gel under non-reducing conditions. The proteins were blotted onto a nitrocellulose membrane and stained as described. The E1 specific antibodies mAb SFV 8/6 [10], [11] and mAb VEE WIS1 as well as the E2-specific antibody mAb 1A3B7 served as positive control and detected proteins of about 47 kDa and 52 KDa. The recombinant antibody recognized specifically the Alphavirus E1 glycoprotein of about 47 kDa (Figure 3A). If the virus sample was additionally reduced by adding DTT prior to SDS-PAGE, no specific binding was observed.

### Neutralisation activity of scFv-Fc fusion proteins against multiple VEEV strains

Heterodimers of the E1 and E2 structural proteins occur on the surface of VEEV and antibody reactivity to either glycoprotein may result in protection against disease *in vitro* and *in vivo* [36], [55], [61]–[63].

For this purpose the ability of scFv-Fc ToR67-3B4 to neutralise virus infectivity *in vitro* was assessed for different VEEV strains in 2 to 3 independent experiments. Serial dilutions of scFv-Fc ToR67 3B4 were mixed with a TCID_50_/ml of 5×10^4^ of VEEV of subtype IA/B and II. The virus antibody mixtures were incubated at 37°C for 2 hours and subsequently subjected to cell culture infection. Residual infectious activity of virus was estimated by specific immunostaining one day post infection with VEEV-specific antibodies. Absorbance (OD450 nm) values obtained with virus samples not preincubated with any antibody served as the positive control and were set as 100% infectivity, non-infected Vero cells were used as the negative control.

A reduction in absorbance at 450 nm equal to or greater than 50% in wells was considered as indicative of neutralization. In addition, virus samples preincubated with VEEV specific antibodies with known neutralising activity, like mAb SFV 8/6 or mAb1A3B7, were used as controls.

ScFv-Fc ToR67-3B4 caused a reduction of infectivity of vaccine strain TC83 to 40% and was able to reduce VEEV infectivity of Everglades virus (formerly subtype II) to 25%. Neutralization of infectivity of both strains was only achieved with high concentrations of antibody. However, scFv-Fc ToR67-3B4 was clearly not able to neutralize the VEEV wildtype strain TrD (Fure 4).

**Figure 4:**
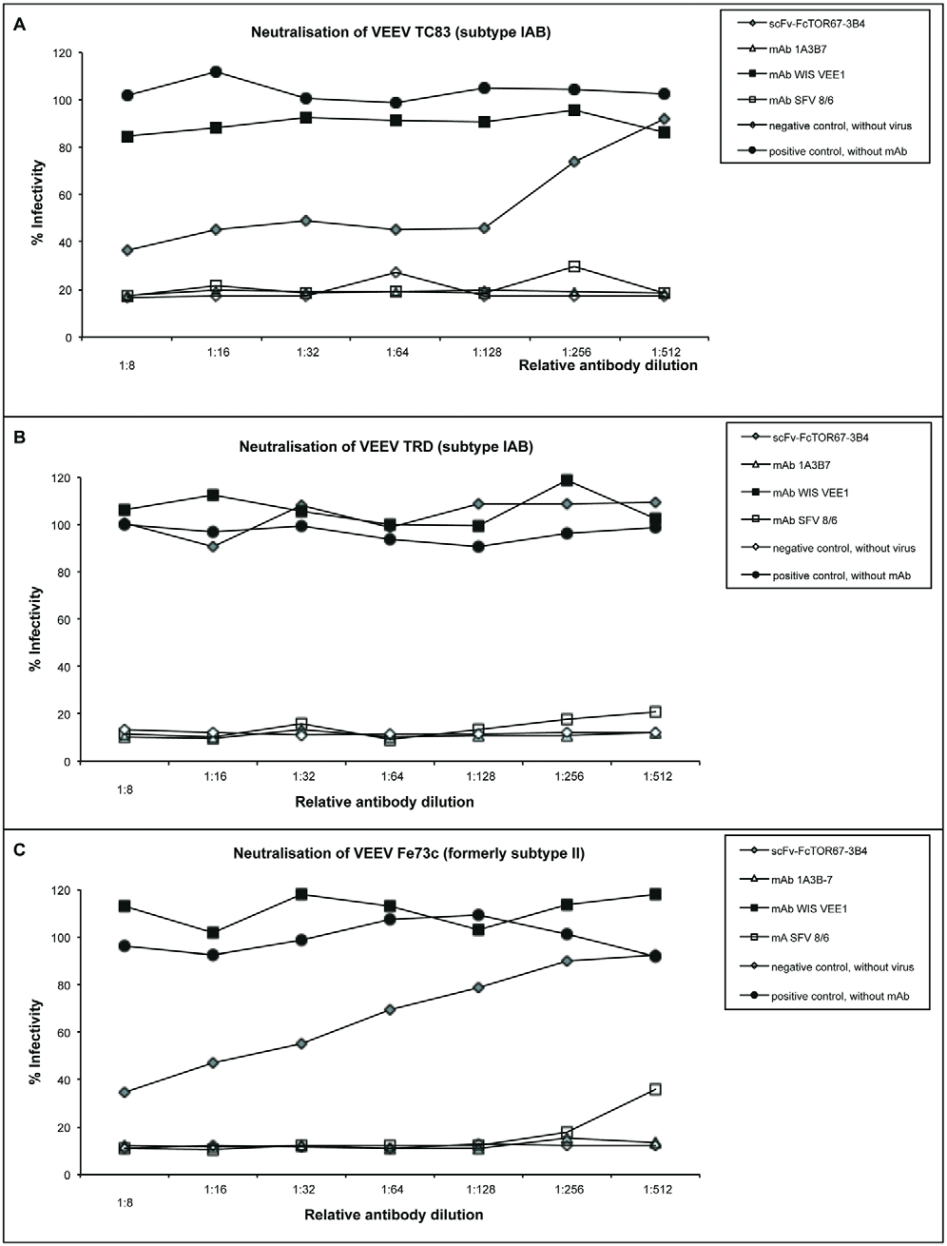
*In vitro* neutralisation activity of scFv-Fc fusion ToR67-3B4 to different VEEV strains and Everglades virus. VEEV strains of subtype IA/B (Panel A and B) and Everglades virus (strain Fe37c, Panel C) with a TCID_50_/ml of 5×10^4^ were incubated with serial dilutions of scFv-Fc ToR67-3B4 and other mAbs at 37°C for 2 hours. Starting concentrations were between 0.5 to 1.0 mg/ml. Subsequently the mixtures were subjected to cell culture infection. Residual infectious activity of virus was estimated by specific immunostaining one day post infection with VEEV-specific antibodies. Absorbance (OD450 nm) values obtained with virus samples not preincubated with any antibody served as positive control and were set as 100% infectivity. Non-infected Vero cells were used as negative control. The mean values of two independent experiments are shown.

Furthermore, the recombinant construct reduced the number of plaques of Mucambo virus (strain BeAn8, formerly subtype IIIA) and plaques of Everglades and Mucambo virus (formerly subtype II and IIIA) produced in the presence of scFv-Fc ToR67-3B4 were greatly reduced in size compared to the controls (data not shown).

### Passive protection of mice *in vivo*

Mice have been widely used for studies of VEE pathogenesis, testing of vaccines and therapeutic treatment. Infection by either the intranasal or subcutaneous routes results in the invasion of the central nervous system and 100% mortality [64]–[66].

Since a sufficient *in vivo* stability of an antibody is a basic requirement for successfull protection *in vivo*, the half-life of ToR67-3B4 in BALB/c mice was estimated before the recombinant antibody was applied in virus challenge experiments. One-hundred µg of the scFv-Fc were injected intraperitoneally and sera were harvested from the murine marginal tail veins at 8 hours, 1, 3, 5, 10, 15 and 25 days post-administration. The serum antibody concentration of the remaining scFv-Fc ToR67-3B4 in blood was determined by ELISA and the recombinant antibody was no longer detectable after 5 days (data not shown). The decrease in antibody serum concentration over time resulted in a half-life of 1.68 d (95% confidence intervals: 1.65–1.71 d, data not shown) that was considered as sufficient to justify analysing the therapeutic potential of this construct.

**Figure 5:**
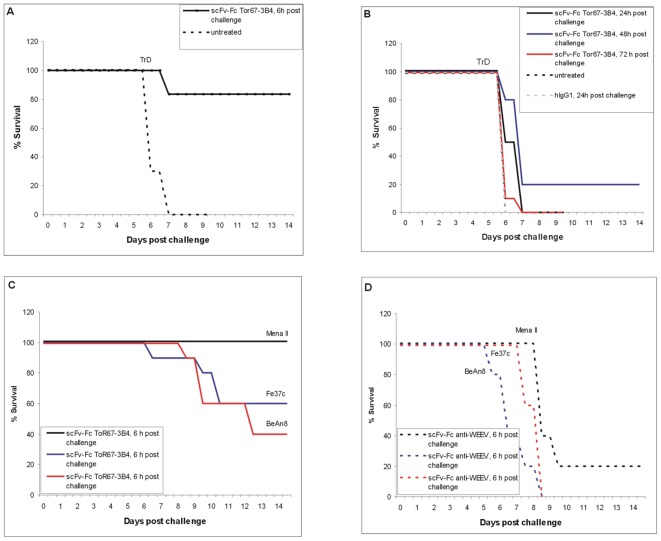
scFv-Fc ToR67-3B4 protects mice against VEEV disease. BALB/c mice were challenged by the aerosol route with approximately 100 LD_50_ VEEV strain TrD (Panel A and B), Mena II, Fe37c and Mucambo virus (BeAn8, Panel C and D). Six, twenty-four, forty-eight and seventy-two hours later they were injected with 100 µg scFv-Fc ToR67-3B4 intraperitoneally (Panel A, B and C, n = 6 or 10). As negative control mice remained either untreated or were injected with a human IgG1 antibody (Panel B) or a nonspecific antibody (anti-WEEV, Panel D, n = 10 or 5). Animals were observed twice daily for clinical signs of infection and were culled when appropriate using humane endpoints.

Therefore, mice were exposed to VEEV strain TrD (subtype IA/B), Mena II (subtype IE), Fe37c (formerly subtype II) and Mucambo virus (strain BeAn8, formerly subtype IIIA) by the aerosol route. Six to seventy-two hours later, 100 µg of scFv-Fc ToR67-3B4 was administered intraperitoneally to a subset of mice and mice were monitored for clinical signs of VEE for 14 days. Naïve mice as well as mice treated with human IgG or a non-specific recombinant antibody (anti-WEEV) did not survive the challenge (median time to death, 6 d) but the recombinant antibody was able to provide significant levels of protection to infected mice (Figure 5) (P = 0.0008, Mantel-Haenszel Logrank test). When the recombinant antibody was administered 6 hours post challenge with VEEV strains TrD or Mena II 80 to 100% of mice survived but died within 6 to 7 days when antibodies were applied after 24 hours or later. Forty to sixty % of mice survived when scFv-Fc ToR67-3B4 was applied 6 hours post challenge with VEEV strains Fe37c and Mucambo virus (strain BeAn8).

In order to ascertain that scFv-Fc ToR67-3B4 was able to prevent viral infection, sera were harvested from the surviving mice and analysed for VEEV-specific antibody by ELISA. A murine-specific peroxidase-conjugated secondary antibody was utilised to distinguish the response induced by the murine immune system from scFv-Fc ToR67-3B4. All mice generated an immune response to VEEV (results not shown), indicating that viral infection had taken place. However, it is not known if this response contributed to the survival of mice treated with scFv-Fc ToR67-3B4.

## Discussion

Screening and isolation of anti-VEEV antibody fragments from a NHP antibody gene library by using phage display was completed successfully. All selected binders shared the same sequence which is not unusual for antibodies from immune libraries containing affinity matured binders.

The scFv as well as the cognate Fc-fusion were able to detect infectious as well as chemically inactivated VEEV viral antigen of subtype IA/B. When applied as detection antibody scFv-Fc ToR67-3B4 exhibited a broad immunological cross-reactivity to all serogroups of the VEEV complex but displayed only marginal cross-reactivity to other Alphavirus species except for EEEV which might be explained by the fact that VEEV is genetically less distinct from EEEV than from SFV and WEEV [1], [67]. In combination with the subtype-specific mAb VEE WIS1 the strain TC83 as representative of VEEV subtype IA/B was detected up to at least aTCID_50_/ml of 10^6^. In contrast, if scFv-Fc ToR67-3B4 was applied as capture antibody, the recombinant antibody displayed a considerably broader cross-reactivity and identified VEEV, EEEV and SFV as well. Furthermore, it showed a low relative binding efficiency to CHIKV and WEEV.

Immunoblot analysis verified that scFv-Fc ToR67-3B4 recognises specifically the viral E1 glycoprotein. We suppose that the Fc-fusion detects a conformational rather than a linear epitope since a specific staining of the viral envelope protein occurred after denaturation at 56°C and 95°C under non-reducing but not under reducing conditions. ScFv-Fc ToR67-3B4 seems to be one of the rare and to our knowledge the first E1-specific scFv-fusion that was able to neutralise virus infectivity *in vitro* for VEEV strains of subtype IA/B, IE, II and Mucambo virus (formerly subtype IIIA). Whereas strains of subtype IA/B are epizootic, strains like Mena II (subtype IE), Fe37c (Everglades virus, formerly subtype II) and BeAn8 (Mucambo virus, formerly subtype IIIA) are enzootic and induce a less progressive disease in mice. Moreover, infection with enzootic strains is considered as non-lethal for human and equidae [70], [71]. Interestingly, scFv-Fc ToR67-3B4 was able to neutralise virus infectivity of vaccine strain TC83 but failed to inactivate the closely related wild type strain TrD although it bound well to it in ELISA. The deduced amino acid sequences of the E1 glycoprotein of strain TC83 and its parent strain TrD differ by a single amino acid change. An introduction of genetic changes manifested at the amino acid level caused by sequentially passaging over years as described [72] was excluded by nucleic acid sequencing.

In the past, antibodies that specifically recognize and neutralise VEEV *in vitro* and *in vivo* have been described. Most of them are directed against the E2 protein, only a few targeted the viral E1 protein [61], [62], [69], [73], [78].

The neutralization efficiency of scFv-Fc ToR67-3B4 was less than that of the two E2-targeting control antibodies mAb SFV 8/6 and 1A3B7. Moreover, incubation of virus with scFv-Fc ToR67-3B4 had an influence on plaque morphology and size. Our data support results of previous studies that clearly documented that neutralisation efficiency for anti-E1 Fabs (fragment, antibody binding) and mAbs are significantly lower than that for anti–E2 mAbs [36], [37], [61]–[63], [74], [75], [79].

Interestingly, scFv-Fc ToR67-3B4 had potent ability to protect mice from infection with VEEV subtype IA/B and IE, Everglades and Mucambo virus (formerly subtypes II and IIIA) when administered 6 hours post challenge but failed to cure mice if applied 24, 48 or 72 hours post challenge. In addition, scFv-Fc ToR67-3B4 was able to protect mice against challenge with VEEV strain TrD although it failed to neutralise this virus strain *in vitro*. Our results give evidence that protection against VEEV disease by scFv-Fc ToR67-3B4 is not necessarily associated with the ability to neutralise VEE virus. The findings reported here are consistent with data from other groups [36], [73], [76]–[78], [80] and we suppose that scFv-Fc ToR67-3B4 binds to a critical site for cell interaction of the VEEV virion either at the level of virus entry, or budding. Whether complement or other Fc effector functions are necessary in VEE virus immunity has to be analysed.

Furthermore, our results clearly indicate that the window in which antibody therapy of VEE is efficacious is short [36], [37], [61], [62], [68]. However, treatment offered a significant benefit for mice as demonstrated in this study. Whether a similar effect in mice is achieved if scFv-Fc ToR67-3B4 is used prophylactically, has to be evaluated. The optimal time for administration of the recombinant binder post challenge seems to be a matter of antibody half-life and clearance. For scFv-Fc ToR67-3B4 the half life was estimated to 1.68 days which is slightly lower than the half-lives of two previously investigated antibodies [68], [69].

Besides applications for specific detection and identification purposes, the NHP antibody described here may have application for human antiviral therapy. Database comparison of scFv ToR67-3B4 indicated the highest similarity to the human IGHV4-59*01 and IGKV2-30*01. Its variable regions were as similar to human V_H or_ V_L_ sequences as 24 and 7% present in Kabat's database respectively. Therefore, scFv ToR67-3B4 might be regarded “as human” as these human sequences. Generally, a high level of humanness is expected to correspond to decreased immunogenicity and could be beneficial for therapeutic treatment of VEE.

It may be even further improved by germline humanisation which aims, without altering the antibody's affinity, at suppressing the macaque specific amino acids [81], [82]. In addition, the human constant regions utilized in the construction of the scFv-Fc could even activate the human immune effectors better than their murine counterparts and possibly allow an even better protection in humans than seen in mice in the present study.

Previously chimeric, humanised and human antibodies have successively been exploited as therapeutics in the murine model but all these antibodies target the VEEV E2 glycoprotein [61], [62], [68], [69]. ToR67-3B4 is to our knowledge the first VEEV inactivating scFv-Fc that targets the VEEV E1 glycoprotein and we assume that the NHP antibody could provide enhanced prophylaxis or immunotherapy for VEEV if used in combination with the so far characterised neutralising E2-specific anti-VEEV antibodies. Moreover, an antibody possessing reactivity to a wide range of VEEV strains *in vivo* may be of benefit as a generic antiviral therapy.
